# Nicotine Induces IL-8 Secretion from Pancreatic Cancer Stroma and Worsens Cancer-Induced Cachexia

**DOI:** 10.3390/cancers12020329

**Published:** 2020-02-01

**Authors:** Patrick W. Underwood, Dong Yu Zhang, Miles E. Cameron, Michael H. Gerber, Daniel Delitto, Michael U. Maduka, Kyle J. Cooper, Song Han, Steven J. Hughes, Sarah M. Judge, Andrew R. Judge, Jose G. Trevino

**Affiliations:** 1 Department of Surgery, College of Medicine, University of Florida Health Science Center, Gainesville, FL 32610, USA; patrick.underwood@surgery.ufl.edu (P.W.U.); dongyu.zhang@surgery.ufl.edu (D.Y.Z.); milesecameron@ufl.edu (M.E.C.); michael.gerber@surgery.ufl.edu (M.H.G.); michael.maduka@surgery.ufl.edu (M.U.M.); kylecooper@ufl.edu (K.J.C.); song.han@surgery.ufl.edu (S.H.); steven.hughes@surgery.ufl.edu (S.J.H.); 2 Department of Surgery, The Sol Goldman Pancreatic Cancer Research Center, The Johns Hopkins University School of Medicine, Baltimore, MD 21287, USA; ddelitt1@jhmi.edu; 3 Department of Physical Therapy, University of Florida Health Science Center, Gainesville, FL 32610, USA; smsenf@phhp.ufl.edu (S.M.J.); arjudge@phhp.ufl.edu (A.R.J.)

**Keywords:** tobacco, smoking, tumor microenvironment, wasting, cytokines, inflammation

## Abstract

Smoking is highly associated with pancreatic cancer. Nicotine, the addictive component of tobacco, is involved in pancreatic cancer tumorigenesis, metastasis, and chemoresistance. This work aimed to describe the role of nicotine within the pancreatic cancer tumor microenvironment. Nicotine treatment was used in vitro to assess its effect on tumor-associated stromal cells and pancreatic cancer cells. Nicotine treatment was then used in a pancreatic cancer patient-derived xenograft model to study the effects in vivo. Nicotine induced secretion of interleukin 8 (IL-8) by tumor-associated stroma cells in an extracellular signal-regulated kinase (ERK)-dependent fashion. The secreted IL-8 and nicotine acted on the pancreatic cancer cell, resulting in upregulation of IL-8 receptor. Nicotine treatment of mice bearing pancreatic cancer patient-derived xenografts had significantly increased tumor mass, increased tumor-free weight loss, and decreased muscle mass. These represent important pathways through which nicotine acts within the tumor microenvironment and worsens pancreatic cancer-induced cachexia, potentially representing future therapeutic targets.

## 1. Introduction

Pancreatic ductal adenocarcinoma (PDAC) is a deadly malignancy with a 5 year overall survival rate of 9% [1]. PDAC has the highest prevalence of cancer-induced cachexia and the highest average weight loss of all malignancies at diagnosis [2]. This contributes to the dismal progress of PDAC. Although overall survival is slowly improving, the many complicated pathways underlying PDAC are now better understood. Smoking, diabetes mellitus, obesity, alcohol use, and pancreatitis are acquired risk factors for the development of PDAC [3]. Tobacco use is one of the most important modifiable risk factors and is attributable to nearly 25% of PDAC cases [4]. The risk of PDAC carcinogenesis decreases upon tobacco cessation and returns to the level of non-smokers after about 20 years [5,6,7]. Continued smoking after surgical resection of PDAC is associated with worse survival [8]. There are many carcinogens in tobacco, and scientists have recently explored their roles in PDAC development.

Nicotine is the addictive component of tobacco. Previous work in murine models has demonstrated that nicotine promotes pancreatic intraepithelial neoplasia (PanIN) lesion development as well as tumor initiation and progression [9,10]. The protein kinase C, Mucin 4 (Muc4), and proto-oncogene tyrosine-protein kinase Src (Src) pathways have all been implicated in promoting the aggressiveness and metastatic potential of PDAC [11,12,13]. Nicotine promotes chemoresistance to gemcitabine in PDAC cells through a Src-dependent pathway [13]. Further work in murine models demonstrated that nicotine-induced gemcitabine resistance was mediated via the Src, Protein kinase b (Akt) and extracellular signal-regulated kinase (ERK) pathways [14]. The summation of this work suggests that nicotine plays a critical role in tumorigenesis, progression, metastasis, and chemoresistance in PDAC.

This previous work has focused on the effects of nicotine in PDAC cells. Up to 80% of PDAC tumor volume is, however, composed of tumor-associated stroma (TAS) [15]. The vast array of signaling that occurs in the tumor microenvironment between TAS and cancer cells has been poorly characterized, and the role of nicotine in TAS is largely unstudied. Our initial investigation of the role of nicotine in TAS has demonstrated that nicotine stimulates hepatocyte growth factor secretion from stromal cells and that this is required for tyrosine-protein kinase Met (c-Met) activation in PDAC [8]. The further effects of nicotine on TAS crosstalk remains unexplored. This work aims to evaluate the effect of nicotine on TAS. Given the severity and degree of cancer-induced cachexia in patients with PDAC, we further aimed to evaluate how nicotine influences systemic wasting. We hypothesized that nicotine acts directly on tumor-associated stromal cells to upregulate local cytokines and further exacerbates an already profoundly cachectic phenotype.

## 2. Results

### 2.1. Nicotine Induced Secretion of IL-8 by Tumor-Associated Stromal Cells

**Figure 1:**
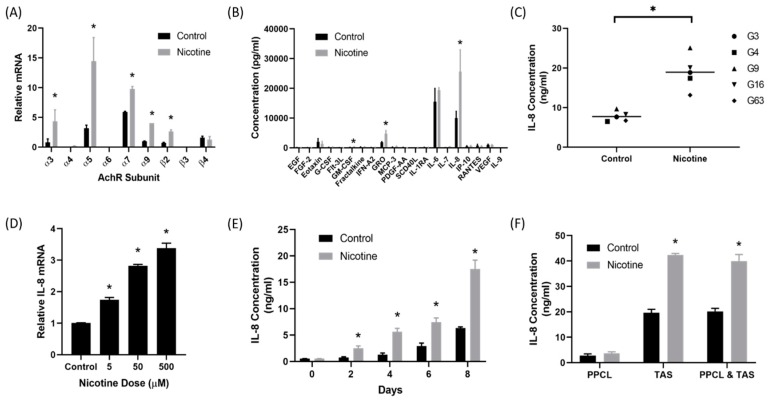
Nicotine induced interleukin 8 (IL-8) secretion from the tumor associated stroma. (**A**) qRT-PCR analysis demonstrated that nicotinic acetylcholine receptor (AchR) subunits were expressed by patient-derived tumor associated stroma (TAS) cells. Treatment of TAS cells with nicotine (50 µM) upregulated (AchR) subunit expression compared to control. (**B**) A 41 protein multiplex assay detected significantly elevated levels of IL-8 secretion from TAS cells treated with 50 µM of nicotine compared to control from three patient-derived TAS cell lines. (**C**) ELISA confirmed that nicotine-treated TAS cells had significantly elevated secretion of IL-8 in five different patient-derived TAS cells. (**D**) IL-8 secretion by TAS cells increased with increased treatment dose. (**E**) IL-8 secretion by TAS cells increased with increased duration of treatment. (**F**) Nicotine-treated primary pancreatic cancer cell lines (PPCL) did not have elevated IL-8 levels. Nicotine-treated TAS cells had significantly elevated IL-8 levels. PPCL and TAS in co-culture had significantly elevated IL-8 secretion.

Quantitative real-time polymerase chain reactions (qRT-PCR) were performed on three TAS cell lines to determine whether the nicotinic acetylcholine receptor (nAChR) was present on TAS cells. There are multiple subtypes of nicotinic acetylcholine receptor with varied functions and roles. Nearly all of the nAChR subunits tested were present on TAS cells (Figure 1A). Forty-eight hours after treatment with nicotine (50 µM), the expression of α3, α5, α7, α9, and β2 were significantly elevated in TAS cells. The α5 nicotinic receptor demonstrated the greatest increase in expression (*p* < 0.05).

Next, a 41 protein multiplex assay was used to identify proteins secreted by TAS cells after nicotine treatment. The multiplex assay detected significant elevations in secretion of granulocyte-macrophage colony-stimulating factor (GM-CSF), growth-regulated oncogene (GRO), and interleukin 8 (IL-8) after treatment with nicotine when compared to control (*p* < 0.05) (Figure 1B). IL-8 demonstrated the most noticeable difference and was secreted at the highest concentrations (*p* = 0.02). Enzyme-linked immunosorbant assays (ELISA) were used to confirm these findings (Figure 1C). Five different patient-derived TAS cell lines were treated with nicotine or control and harvested 48 h after treatment. IL-8 secretion was significantly higher in nicotine treated TAS cell lines (18.9 ± 4.32 vs. 7.7 ± 1.28 ng/mL, *p* < 0.001).

qRT-PCR demonstrated that nicotine treatment of TAS cells increased IL-8 transcription in a dose-dependent manner (*p* < 0.0001) (Figure 1D). Further analysis using ELISA demonstrated that TAS cells treated with 50 µM of nicotine every two days secreted increasing concentrations of IL-8 in a time-dependent manner (*p* < 0.01) (Figure 1E). In order to fully understand IL-8 secretion in the tumor microenvironment, patient-derived, primary pancreatic cancer cell lines (PPCL) and TAS cells were cultured separately and in co-culture. Cells were treated with nicotine (50 µM) or control (phosphate buffered saline) and harvested 48 h after treatment (Figure 1F). PPCL levels had low levels of IL-8 secretion before and after treatment with nicotine (2.8 ± 0.67 ng/mL and 3.7 ± 0.54 ng/mL, *p* = 0.27). As expected, TAS cells demonstrated significantly increased IL-8 secretion in nicotine-treated cells (19.6 ± 1.32 ng/mL vs. 42.4 ± 0.60 ng/mL, *p* = 0.002). TAS and PPCL cells in co-culture demonstrated a similar increase in IL-8 secretion in cells treated with nicotine (20.2 ± 1.17 ng/mL vs. 40.0 ± 2.58 ng/mL, *p* = 0.01).

### 2.2. Secretion of IL-8 in Nicotine-Treated Tumor-Associated Stroma Cells Was Dependent on the ERK Pathway

**Figure 2:**
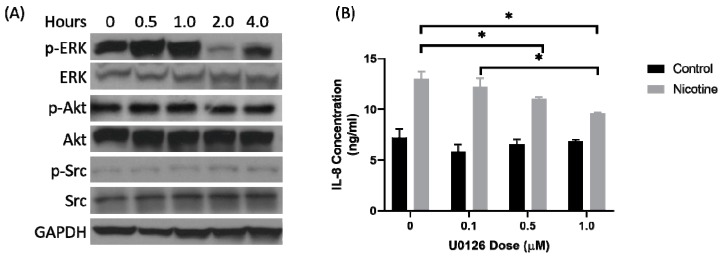
Secretion of IL-8 by the tumor-associated stroma was extracellular signal-regulated kinases (ERK) dependent. (**A**) Western blot analysis of TAS cells treated with nicotine (50 µM) for the indicated time demonstrated phosphorylation, signified by “p-“, of ERK but no change in protein kinase B (Akt) or Proto-oncogene tyrosine-protein kinase Src (Src). (**B**) TAS cells were then pre-treated with an escalating dose of U0126, an inhibitor of MAPK/ERK kinase (MEK) that phosphorylates ERK, and then with nicotine or control 1 h later. MEK inhibition decreased IL-8 secretion in the nicotine-treated TAS cells. IL-8 secretion decreased with an increasing dose of U0126. The uncropped blots and molecular weight markers are shown in Figure S1 and Table S1.

ERK, Src, and Akt are involved in the nicotine signaling in PDAC cells [14]. Patient-derived TAS cells were treated with nicotine (50 µM) and harvested at time intervals after treatment. Western blot was used to assess phosphorylation of ERK, Src, and Akt (Figure 2A). There was increased phosphorylation of ERK for 1 h after treatment with nicotine but no change in phosphorylation of Akt or Src. Next, a MAPK/ERK kinase (MEK) inhibitor, U0126, was used 1 h prior to nicotine treatment at increasing doses (Figure 2B). Inhibition of MEK blocked the phosphorylation of ERK. Control-treated TAS cells demonstrated no significant differences in IL-8 secretion with increasing doses of U0126. In nicotine-treated TAS cells, however, U0126 significantly decreased IL-8 secretion as the dose was increased (*p* < 0.001). TAS cells treated with nicotine were also assessed for activation of the Src and Akt pathway (Figure 2A). Src or Akt phosphorylation was not affected by nicotine treatment.

### 2.3. There Was Upregulation of IL-8 Receptors on Pancreatic Cancer Cells After Treatment with Nicotine

**Figure 3:**
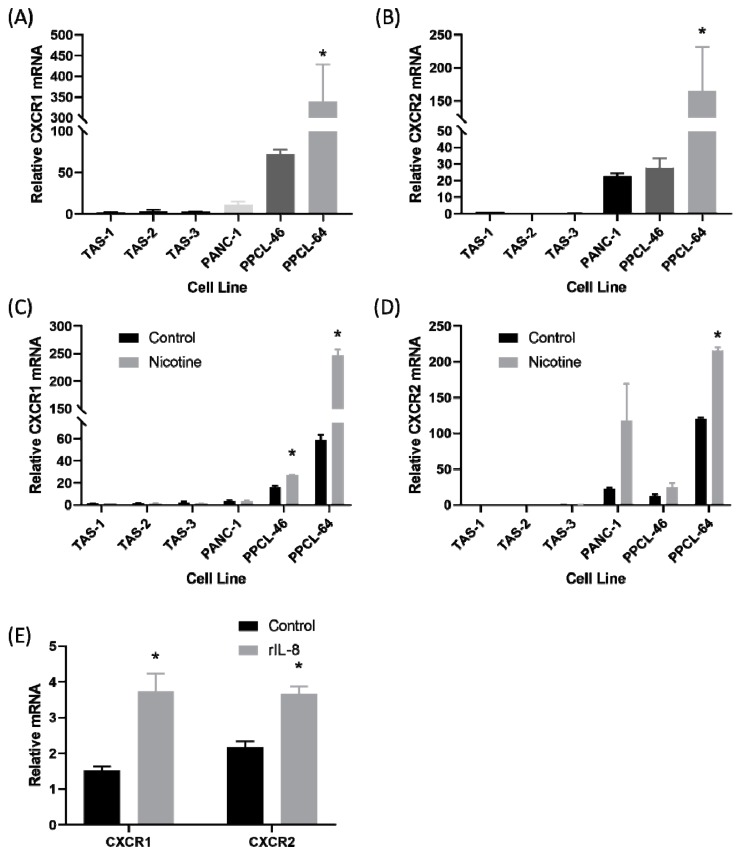
The IL-8 receptor was upregulated on pancreatic cancer cells lines in response to nicotine treatment and recombinant IL-8 (rIL-8) treatment. (**A**) Expression of the C-X-C motif chemokine receptor-1 (CXCR1) receptor, measured by qRT-PCR, was significantly elevated in patient-derived primary pancreatic cancer cell line (PPCL-64) compared to PPCL-46, PANC-1, and patient-derived tumor-associated stromal cell lines. (**B**) Similarly, expression of the C-X-C motif chemokine receptor-2 (CXCR2) receptor was significantly elevated in PPCL-64 compared to the other cell lines. (**C**) Nicotine treatment significantly increased CXCR1 expression in PPCL-64 and PPCL-46, but not PANC-1 or TAS cells. (**D**) Expression of the CXCR2 receptor was significantly elevated in PPCL-64 cells treated with nicotine compared to other cells lines. (**E**) Treatment of PPCL-46 with 1.25 nM recombinant IL-8 for 24 h resulted in a significant increase in CXCR1 and CXCR2 messenger RNA (mRNA).

Il-8 acted as a ligand on the C-X-C motif chemokine receptor-1 (CXCR1) and C-X-C motif chemokine receptor-2 (CXCR2). Expression of CXCR1 and CXCR2 was evaluated with qRT-PCR in three patient-derived TAS cell lines, two PPCL, and PANC-1 cells (Figure 3A,B). The two patient-derived cell lines, PPCL-46 and PPCL-64, demonstrated the highest relative expression of CXCR1, whereas very little expression was observed in TAS cell lines (*p* < 0.001). Similarly, CXCR2 relative expression was higher in the two patient-derived PPCL and PANC-1. CXCR2 expression was also very low in TAS cell lines (*p* = 0.002). Next, cell lines were treated with either nicotine (50 µM) or control (phosphate-buffered saline) and harvested at 24 h (Figure 3C,D). CXCR1 messenger RNA (mRNA) expression was significantly elevated in nicotine-treated PPCL-46 and PPCL-64 compared to control-treated cells (*p* < 0.05). CXCR2 expression was significantly elevated in nicotine-treated PPCL-64 (*p* < 0.05). Similarly, CXCR2 mRNA expression was generally increased in nicotine-treated PANC-1 and PPCL-46 (*p* = 0.12 and *p* = 0.10, respectively). Finally, PPCL were treated with 1.25 nm of recombinant IL-8 (rIL-8) or control for 24 h and harvested (Figure 3E). There was a significant increase in CXCR1 (*p* = 0.026) and CXCR2 expression (*p* = 0.016) after treatment with rIL-8, suggesting autocrine regulation.

### 2.4. PDAC-Bearing Mice Treated with Nicotine Had Worsened Cancer-Induced Cachexia

**Figure 4:**
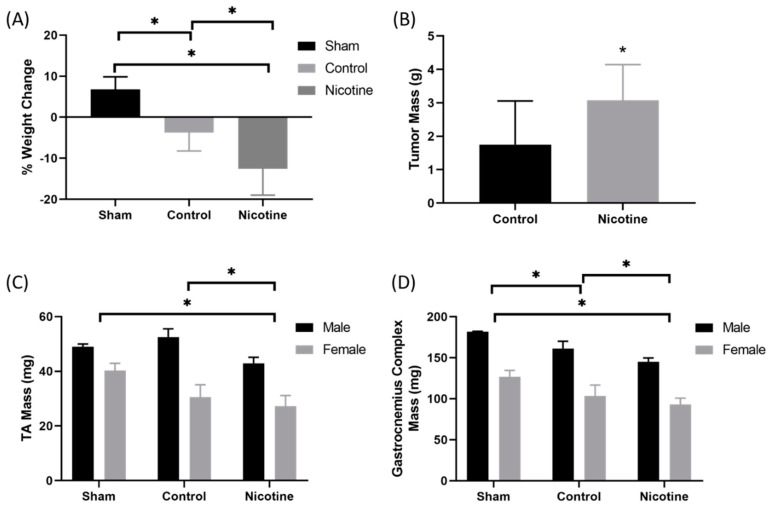
Nicotine treatment in mice bearing orthotopic patient-derived xenografts from pancreatic ductal adenocarcinoma increased cachexia. (**A**) Patient-derived xenografts from patients with pancreatic ductal adenocarcinoma were implanted on the pancreata of NOD.Cg-Prkdc^scid^Il2rg^tm1Wjl^/SzJ (NSG) mice at 90 days of life. Intraperitoneal injections of 1 mg/kg nicotine or control (PBS) were given 3 days a week for 6 weeks. At endpoint, tumor-bearing mice treated with nicotine lost significantly more tumor-free weight than control and sham mice. (**B**) Nicotine-treated mice had larger tumors on average than control. (**C**) The tibialis anterior and (**D**) gastrocnemius complex muscles of the tumor-bearing nicotine-treated mice weighed significantly less than control-treated mice and sham mice.

In order to assess the effect of nicotine treatment on mice bearing patient-derived pancreatic cancer xenograft, two sex-matched patient-derived xenografts from patients with PDAC were implanted onto the pancreas of 12 week old male and female NOD.Cg-Prkdc^scid^Il2rg^tm1Wjl^/SzJ (NSG) mice. Previous work by our lab has shown that orthotopic patient-derived pancreatic cancer xenografts recapitulate human disease and induce cachexia in mice [15]. Beginning 14 days after implantation, mice were treated with intraperitoneal injection of 1 mg/kg of nicotine or control (PBS) 3 days per week for 6 weeks. At endpoint, mice were euthanized and weight change, tumor mass, and skeletal muscle mass were measured. At endpoint, there were three males and six females in the treatment group, six males and six females in the control group, and two males and three females in the sham group. Three male mice in the treatment group died. Mice treated with nicotine lost significantly more tumor-free weight (−12.5 ± 6.4%) throughout the experiment compared to control-treated mice (−3.8 ± 4.5%, *p* = 0.0024). Mice undergoing sham surgery gained weight (6.8 ± 3.1%) throughout the experiment (Figure 4A). Consistent with other work demonstrating tumor progression with nicotine treatment [12], tumor mass was significantly elevated in nicotine-treated mice compared to control (3.08 ± 1.07 g vs. 1.75 ± 1.31 g, *p* = 0.02) (Figure 4B).

Next, limb muscle mass was evaluated. Mice were grouped by sex. Male (M) and female (F) nicotine-treated mice had significantly lower tibialis anterior (TA) mass (M = 42.9 ± 2.2 mg, *F* = 27.3 ± 3.8 mg) compared to control-treated (M = 52.5 ± 3.0 mg, *F* = 30.5 ± 4.6 mg, *p* = 0.002) and sham mice (M = 49.0 ± 1.0 mg, *F* = 40.3 ± 2.6 mg, *p* < 0.001) (Figure 4C). Similarly, male and female nicotine-treated mice had significantly lower gastrocnemius complex mass (M = 145.1 ± 3.8 mg, *F* = 93.1 ± 7.0 mg) compared to control-treated (M = 161.2 ± 8.0 mg, *F* = 103.4 mg, *p* = 0.014) and sham mice (M = 181.9 ± 0.25 mg, *F* = 126.9 ± 6.2 mg, *p* < 0.001) (Figure 4D).

## 3. Discussion

**Figure 5:**
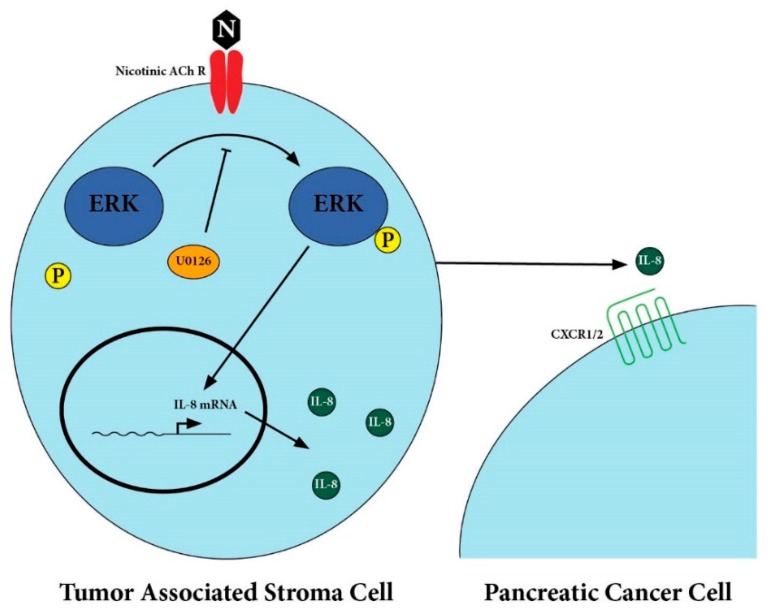
Proposed pathway for IL-8 secretion by tumor-associated stromal cells.

Smoking is strongly associated with pancreatic cancer and smoking cessation is the most effective modifiable behavior to prevent pancreatic cancer development. Previous work has demonstrated a critical role of nicotine in PDAC tumorigenesis, progression, and metastasis. The importance of this observation is amplified by the growing use of e-cigarettes and vaping products, which can contain higher levels of nicotine than traditional tobacco products [16]. This work is an initial step towards understanding how nicotine acts within the tumor microenvironment of PDAC. Nicotine acts on nicotinic receptors present on TAS cells to induce secretion of IL-8 in an ERK-dependent pathway, as depicted in Figure 5. Given that IL-8 levels are known to be associated with degree of cancer-induced cachexia for a number of malignancies including PDAC, we next sought out to identify an association between nicotine treatment and cancer-induced cachexia [17,18,19,20]. We demonstrated that nicotine-treated tumor bearing mice had significantly increased tumor mass, tumor-free weight loss, and decreased skeletal muscle mass. This work identified a potentially crucial link between nicotine, the PDAC tumor microenvironment, and cancer-induced cachexia.

Nicotine treatment results in increased expression of the nicotinic acetylcholine receptor subunits on TAS cells increased secretion of IL-8. The IL-8 secretion was ERK-dependent. This was associated with worsened cachexia in a patient-derived xenograft murine model.

Importantly, a number of other studies have demonstrated the effects of nicotine on IL-8 secretion by cancer cells. Oral epidermoid carcinoma cells and oral squamous cell carcinoma cells are induced to secrete IL-8 by nicotine [21,22]. Similarly, nicotine has been shown to induce IL-8 production by periodontal ligament cells upon binding the α7 nAChR [23]. Paralleling our work, nicotine has been shown to act on various nAChRs in an ERK-dependent pathway to stimulate IL-8 production by macrophages in the lungs [24]. Furthermore, nicotine induces IL-8 secretion by human bronchial epithelial cells and neutrophils in lung parenchyma [25,26]. These studies have focused on the local effects on nicotine in the respiratory tract, but given its systemic absorption and distribution, nicotine has effects on all body tissues. A link between IL-8 levels in the tumor microenvironment of smokers with PDAC represents a critical next step to further understanding the role of smoking within the tumor microenvironment.

The cytokine IL-8 is produced by muscle, adipose, macrophages, and epithelial cells. More recently, it has been shown to be involved in tumor growth and progression in a number of cancers [27,28,29]. IL-8 is associated with cancer-induced cachexia. This devastating condition is characterized by muscle and adipose wasting. It leads to decreased quality of life and poorer survival in patients with PDAC [8]. Patients with PDAC have the highest rates of cancer-induced cachexia [2]. Elevated serum IL-8 correlates with cancer-induced cachexia and poor outcomes in patients with PDAC [17]. IL-8 levels are also associated with cachexia in prostate and gastroesophageal cancers [19,20]. Certain genetic polymorphisms of IL-8 found in gastric cancer are associated with cachexia [18,30]. We found that nicotine-treated tumor-bearing mice had significantly worsened cancer-cachexia compared to controls. This represents an important finding, as nicotine may be contributing to worsened cancer-induced cachexia, and ultimately worse survival.

Our study had a number of important limitations. All of this work was carried out in cell and murine models. Work to demonstrate these effects in smokers with PDAC is an important next step. Further, although mice treated with nicotine had worsened cancer-induced cachexia, we did not establish a direct link with IL-8. This observation could be due to elevated IL-8 levels transiently in the serum, or more likely, due to elevated IL-8 levels within the muscle itself. All PDAC tumors are not the same. Although we used multiple patient-derived cell lines and patient-derived xenografts, these models may not apply to all individuals. The degrees to which nicotine affects the microenvironment likely varies by individual.

In conclusion, we report that nicotine has direct effects on pancreatic cancer tumor-associated stroma. Nicotine induces IL-8 secretion by the TAS and upregulates the IL-8 receptor on pancreatic cancer cells. In vivo, nicotine increases tumor burden and worsens systemic wasting. Further work is needed to better characterize this pathway, observe its role in the tumor microenvironment, and develop specific therapies that can target the many pathways of pancreatic cancer.

## 4. Materials and Methods

### 4.1. Cell Culture and Reagents

Consent for tissue acquisition was obtained from all patients to establish primary cancer cell lines (PPCL), patient-derived tumor-associated stroma (TAS) cell lines, and the patient-derived xenograft (PDX) models noted below. This was approved by the Institutional Review Board at the University of Florida (IRB 201600873). The PPCL were established and authenticated from PDX using previously described techniques [31]. Commercially available PANC-1 cells were cultured for experiments where noted. TAS cell lines were established from surgically resected PDAC tissue using methods previously described [32]. Cells were cultured in Dulbecco’s modified Eagle’s medium/F12 (DMEM/F12) with 10% fetal bovine serum (FBS; Atlanta Biologicals, Atlanta, GA, USA) and antibiotic/antimycotic solution (Corning Inc., Corning, NY, USA) at 5% CO_2_/95% air at 37 °C. For experiments, 500,000 cells were seeded onto a 10 cm plate and incubated for 24 h. The media was changed to serum-free media with or without the indicated drug and treated for the indicated time. Nicotine (Sigma Aldrich, St. Louis, MO, USA) dose was 50 µM. For cell treatment with recombinant IL-8 (R&D Systems, Minneapolis, MN, USA), the dose was 1.25 nM. Experiments evaluating MEK inhibition were performed with U0126 (Cell Signaling Technologies, Danvers, MA, USA). Cells were pre-treated with the MEK inhibitor, and nicotine treatment began 1 h later.

### 4.2. Quantitative RT-PCR

Cells from culture were collected. RNA was extracted using RNeasy Mini (QIAGEN, Valencia, CA, USA). A total of 100 ng of RNA was used for reverse transcription. qRT-PCR was performed using iScript One-Step RT-PCR kit with SYBR Green (BIO-RAD, Hercules, CA, USA). A total volume of 25 µL was used for each reaction. RNA was probed for AchR subunits, IL-8, CXCR1, or CXCR2 mRNA. All data were normalized to Glyceraldehyde 3-phosphate dehydrogenase (GAPDH) expression. Primers: CXCR1 forward 5′-CTCCTACTGTTGGACCACC-3′, reverse 5′-CACTAGGGCATAGGCGATGAT-3′; CXCR2 forward 5′-ATCGGTGGCCACTCCAATAAC-3′, reverse 5′-TAAATCCTGACTGGGTCGCTG-3′; IL-8 forward 5′-TGGCAGCCTTCCTGATTTCTG-3′, reverse 5′-AACCCTCTGCACCCAGTTTTC-3′; GAPDH forward 5′-CTGACTTCAACAGCGACACC-3′, reverse 5′- AGCCAAATTCGTTGTCATACC-3′; Acetylcholine receptor (AchR)α3 forward 5′-AACCTGTGGCTCAAGCAAATCT-3′, reverse 5′-CATGAACTCTGCCCCACCAT-3′; AchRα4 forward 5′-GTGGATGAGAAGAACCAGATGATG-3′, reverse 5′-CAGCGCAGCTTGTAGTCGTG-3′; AchRα5 forward 5′-AGATGGAACCCTGATGACTATGGT-3′, reverse 5′-AAACGTCCATCTGCATTATCAAAC-3′; AchRα6 forward 5′-TGGCCAACGTGGATGAAGTAA-3′, reverse 5′-TCTCAATGCCATCATATTCCATTG-3′; AchRα7 forward 5′-GCTGCTCGTGGCTGAGATC-3′, reverse 5′-TGGCGAAGTACTGGGCTATCA-3′; AchRα9 forward 5′-AAAGATGAACTGGTCCCATTCCT-3′, reverse 5′-AAGGTCATTAAACAACTTCTGAGCATAT-3′; AchRβ2 forward 5′-CTGGATCCTTCCCGCTACAAC-3′, reverse 5′-TGGGTCAGCCAGACATTGGT-3′; AchRβ3 forward 5′-AACAGTTCCGTTTGATTTCACGAT-3′, reverse 5′-CAGCCAGGTAGTACAAGACTGGAAAT-3′; AchRβ4 forward 5′-TCACAGCTCATCTCCATCAAGCT-3′, reverse 5′-CCTGTTTCAGCCAGACATTGGT-3′.

### 4.3. ELISA and 41-Plex Protein Assay

Supernatants from cell culture were collected after treatment with or without nicotine for 48 h as noted above. For the multiplex protein assay, supernatants and assay were prepared per the manufacturer protocol (HCYTMAG-60K-PX41, Millipore Sigma, Burlington, MA, USA). A total of 25 µL of supernatant was used in each well. Data were acquired using the MAGPIX system (Luminex Corporation, Austin, TX, USA) and analyzed using MILLIPLEX Analyst 5.1 (Millipore Sigma, Burlington, MA, USA). For ELISA, supernatants and reagents were added to the 96-well plate per the manufacturer’s protocol (50-246-338, Fisher Scientific, Hampton, NH, USA). The optical density was read at 450 nm.

### 4.4. Western Blot Analysis

Cells were collected from culture and lysed with RIPA lysis buffer (Santa Cruz Biotechnology, Dallas, TX, USA). Total protein was analyzed using the DC Protein Assay (BIO-RAD, Hercules, CA, USA). Proteins were denatured at 95 °C for 5 min. A total of 25 µg of total protein was loaded in each lane. After running, the gel was transferred to polyvinylidene fluoride (PVDF) membrane using the Trans-Blot Turbo Transfer System (BIO-Rad, Hercules, CA, USA). Membranes were probed with total and phospho-ERK, -Akt, or -Src primary antibodies (Cell Signaling Technologies, Danvers, MA, USA).

### 4.5. Patient-Derived Xenografts

All animal studies were approved by the University of Florida Institutional Animal Care and Use Committee (IACUC 201706590). Methods of our PDX model have previously been described [15]. For this work, 2 × 2 mm sections of PDAC PDX were implanted onto the pancreas of sex-matched NOD.Cg-Prkdc^scid^Il2rg^tm1Wjl^/SzJ (NSG) 12 week old mice (Jackson Laboratory, Bar Harbor, ME, USA). Two PDX were used for these experiments, one male and one female. The male tumor, G160p3, was derived from a non-Hispanic, White male who underwent a pancreaticoduodenectomy for T1N1, poorly differentiated PDAC. G171p3 was derived from a non-Hispanic, White female who underwent a distal pancreatectomy and splenectomy for T3N2, poorly differentiated PDAC. Fourteen days after implantation, injections began with 1 mg/kg of nicotine or an equivalent volume of control (PBS) 3 days per week for 6 weeks. Three male mice in the treatment group died before harvest and were not included in the final analysis. There were three males and six females in the treatment group, six males and six females in the control group, and two males and three females in the sham group on final analysis. After 6 weeks of treatment, mice were weighed and euthanized. Tumors, left tibialias anterior muscle, and left gastrocnemius complex were weighed and collected.

### 4.6. Statistical Analysis

All statistical analyses were carried out in SPSS version 25 (IBM SPSS Statistics for Windows; IBM Corp). Figures were created in GraphPad Prism 7 (San Diego, CA, USA). Experiments comparing treatment to control groups were compared using independent samples *t*-test. Analysis of variance (ANOVA) with multiple comparisons were used for experiments comparing multiple groups. A *p*-value of <0.05 was considered significant.

## 5. Conclusions

Smoking is a leading cause of pancreatic cancer. Nicotine acts on stromal cells in the tumor microenvironment to secrete IL-8, which subsequently acts on the pancreatic cancer cells. Nicotine treatment of mice is associated with worsened cancer cachexia. This work highlights the importance of smoking cessation in patients with PDAC or at risk of developing PDAC. It further describes new pathways that may help us understand the complex interactions within the tumor microenvironment, providing possible pathways to exploit in future therapeutics. Continued work into understanding how smoking contributes to tumorigenesis, metastasis, and cancer-induced cachexia is necessary to understand the process of this devastating disease.

## Supplementary Materials

The following are available online at https://www.mdpi.com/2072-6694/12/2/329/s1, Figure S1: Original western blots, Table S1: Densitometry of each band normalized to GAPDH.
