# Supplementary material for: Nicotine Induces IL-8 Secretion from Pancreatic Cancer Stroma and Worsens Cancer-Induced Cachexia
Source: Cancers (Basel). 2020 Feb 1;12(2):329. doi: 10.3390/cancers12020329 (PMC7072641; doi:10.3390/cancers12020329)
Supplement: Supplementary file 1 [file cancers-12-00329-s001.pdf]

Supplementary Materials: Nicotine Induces IL-8 Secretion from Pancreatic Cancer Stroma and Worsens Cancer-Induced Cachexia

Patrick W. Underwood, DongYu Zhang, Miles E. Cameron, Michael H. Gerber, Daniel Delitto, Michael U. Maduka, Kyle J. Cooper, Song Han, Steven J. Hughes, Sarah M. Judge, Andrew R. Judge and Jose G. Trevino

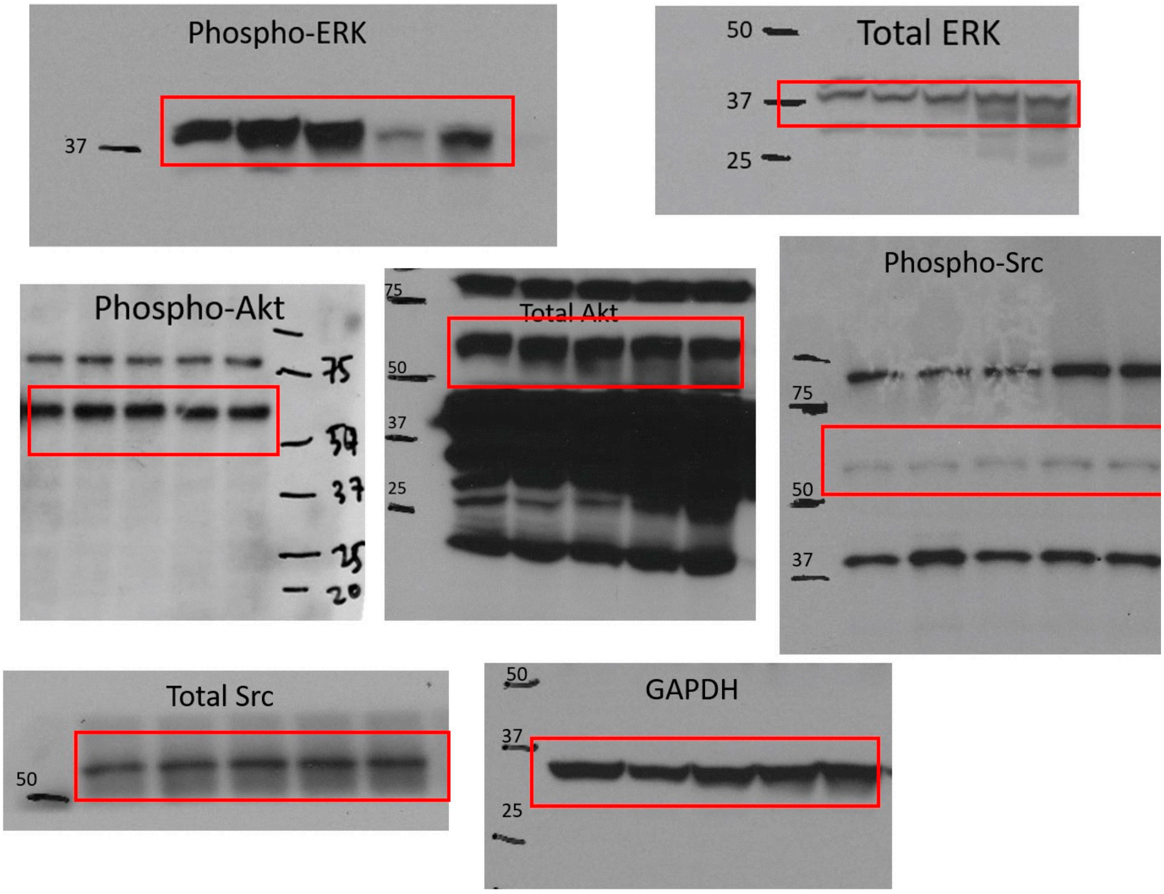

Figure S1. Original western blots.

Table S1. Densitometry of each band normalized to GAPDH.

Hours	0	0.5	1.0	2.0	4.0
Phosph-ERK	0.9032	1.6154	1.1337	0.2405	0.3159
Total ERK	0.0797	0.1434	0.1233	0.1215	0.1047
Phospho-Akt	0.3940	0.6455	0.4376	0.4000	0.4000
Total AKT	1.1060	1.0769	0.6782	0.7950	1.0560
Phospho-Src	0.0412	0.0599	0.0609	0.0945	0.0672
Total Src	0.2018	0.1378	0.1569	0.1665	0.2233

© 2020 by the authors. Licensee MDPI, Basel, Switzerland. This article is an open access article distributed under the terms and conditions of the Creative Commons Attribution (CC BY) license (http://creativecommons.org/licenses/by/4.0/).
